# The Funds of Trade Unions

**Published:** 1913-09-06

**Authors:** Percy C. Raiment

**Affiliations:** 69 Brook Green, London, W.


### THE NATIONAL MEDICAL GUILD.

President: CHAS. BUTTAR, M.A., M.D., B.C.

Gen. Sec.: P. C. RAIMENT, M.R.C.S., L.R.C.P.

Offices: 34 Villiers Street, Strand, London, W.C.

### The Funds of Trade Unions.

*To the Editor of* THE HOSPITAL.

DEAR SIR,—I have read with much interest the controversy that has been proceeding between Dr. Fothergill and the National Medical Guild, and as Secretary of that body, may I be allowed to add a few words? It must be understood that the remarks that I am about to make are my personal views, and are not necessarily shared by the other officials of the Guild.

There is one fact that stands out clearly above all the dust of controversy, and one that I have never yet seen answered, even by Dr. Fothergill, and it is this. The funds of a trade union are exempt from any fear of attachment whilst the funds of any other organisation are in danger of it. That is the reason why I personally am a trade unionist, and, until I can find a body that can obtain the same exemptions that the Guild can, I shall not only remain a member, but I shall continue to endeavour to persuade others to join us. As to the British Medical Association, I am, and I hope I always shall remain a member, but at the same time I must point out to them that they are proposing to engage in a scheme that will inevitably, in the hour of trial, be found wanting, and the profession once again driven to accept anything that is offered to them because their organisation has no funds to fight with.—I am, Dear Sir, yours, etc.,

PERCY C. RAIMENT.

69 Brook Green, London, W.
August 30, 1913.

